# Evaluating Epicardial Fat Density Using ROI-Based Analysis: A Feasibility Study

**DOI:** 10.3390/jcdd12030081

**Published:** 2025-02-20

**Authors:** Giovanni Lorusso, Nicola Maggialetti, Luca De Marco, Sterpeta Guerra, Ilaria Villanova, Sara Greco, Chiara Morelli, Nicola Maria Lucarelli, Michele Mariano, Amato Antonio Stabile Ianora

**Affiliations:** Section of Radiology and Radiation Oncology, Interdisciplinary Department of Medicine, University of Bari “Aldo Moro”, 70124 Bari, Italyn.maggialetti@gmail.com (N.M.); s.guerra91@tiscali.it (S.G.); ilaria.villanova05@gmail.com (I.V.); saragrecosa@gmail.com (S.G.); dottchiaramorelli@gmail.com (C.M.); nicola.lucarelli@policlinico.ba.it (N.M.L.); marmic37@libero.it (M.M.); amatoantonio.stabileianora@uniba.it (A.A.S.I.)

**Keywords:** cardiac computed tomography (CCT), epicardial adipose tissue (EAT), epicardial fat tissue (EFT), epicardial fat volume (EFV), epicardial fat density (EFD), region of interest (ROI)

## Abstract

Epicardial fat density (EFD) is implicated in cardiovascular diseases. This study aimed to assess the regional variability of epicardial fat density (EFD) using coronary computed tomography (CCT) and evaluate the feasibility of ROI-based measurements as an alternative to full segmentation. A retrospective analysis was conducted on 171 patients undergoing coronary CCT. EFD was measured on non-contrast scans acquired globally and in three predefined regions of interest (ROIs) for coronary calcium scoring: the aortic bulb, right posterolateral wall, and cardiac apex. Global EFD was quantified using semi-automated segmentation software (3D Slicer 5.6.2), while regional EFD values were manually determined. Statistical analyses were performed to compare global and regional EFD measurements. Global EFD averaged −83.92 ± 5.19 HU, while regional EFD showed significant variability. The aortic bulb had lower EFD values (−97.54 ± 12.80 HU) compared to the apex (−93.42 ± 18.94 HU) and right posterolateral wall (−94.99 ± 12.16 HU). Paired *t*-tests confirmed statistically significant differences between global and regional EFD values (*p* < 0.000). This study highlights significant regional variability in EFD across specific cardiac regions, suggesting that ROI-based assessments may not reliably reflect global EFD characteristics.

## 1. Introduction

The adipose tissue of the heart is divided into two different layers: pericardial adipose tissue (PAT), located in the parietal layer of the pericardium, and epicardial adipose tissue (EAT), also referred to as epicardial fat tissue (EFT). EAT is a particular type of adipose tissue located around the heart, between the myocardium and the visceral layer of the pericardium [1,2,3]. Each layer has a different embryological origin and its own vascularization [4]; unlike PAT, EAT originates from the splanchnopleuric mesoderm, the same embryonic layer that gives rise to mesenteric and omental fat [5].

EAT is metabolically active and produces a series of pro-inflammatory molecules like cytokines (TNF-alpha, IL-6, monocyte chemoattractant protein-1) and adipocytokines which can have direct effects on the coronary vessels and on the myocardium itself [6,7,8]. These substances have protective and dangerous effects. EAT serves as a local energy reservoir, as mechanical protection for coronary vessels, and contributes to thermoregulation of the heart, particularly in metabolic stress. Furthermore, it is an immune barrier and protection of the coronary arteries; it also responds to energy needs, as it has a regulatory role in the release and absorption of free fatty acids.

However, EAT also has important implications in cardiovascular pathology. EAT’s proximity to coronary arteries and the myocardium also enables direct paracrine interactions that can influence cardiovascular health. Its secretion of adipokines, inflammatory mediators, and free fatty acids may induce atherosclerosis and promote myocardial inflammation [9]. It has been demonstrated that an increase in epicardial fat volume (EFV) leads to hypoxia and dysfunction, characterized by enhanced lipolysis and consequent inflammation, resulting in alterations to the metabolic profile; when this dysfunction occurs, it may contribute to the development of coronary artery disease (CAD).

Numerous studies [10,11,12,13] demonstrate the role of EAT in the development of heart failure, atrial fibrillation, metabolic syndrome, and other cardiovascular disorders.

In this context, quantitative imaging techniques are indispensable for assessing EAT to potentially establish it as a non-invasive marker for cardiovascular risk.

While the overall EFV has been shown to correlate with cardiovascular risk, recent attention has shifted toward understanding the role of epicardial fat density (EFD), a measure of fat attenuation on CT scans. EFD reflects the composition of the fat, with higher density values indicating fat infiltration by connective tissue, fibrosis, or inflammatory cells [14]. These compositional changes may be more closely related to cardiovascular disease than fat volume alone [15,16].

The primary methods used for assessing EAT include echocardiography, cardiac computed tomography (CCT), and cardiac magnetic resonance (CMR). The quantification of EAT using CCT volumetric technique permits EFD measurement, but it is hindered by cost, technical expertise, time constraints, and lengthy segmentation times. Accurate measurements require numerous images with thin slice-thickness and specific segmentation tools which are not currently widespread; that significantly limits its feasibility for routine clinical practice [17].

This study aimed to explore whether EFD could be assessed without the use of specific segmentation software by comparing EFD values measured across the entire EAT and in three specific regions of interest (ROI)—the aortic bulb, right posterolateral wall, and cardiac apex. The ROI-based approach not only could improve feasibility by reducing processing time but also allow for targeted analysis of specific myocardial regions, which may better capture localized variations in EFD that are relevant for cardiovascular risk assessment.

## 2. Materials and Methods

### 2.1. Study Population

This study was conducted in alignment with ethical principles, adhering to the Declaration of Helsinki.

This retrospective study analyzed data from 200 patients referred to our institution for coronary CT angiography (CCTA) between January 2024 and October 2024, to explore whether EFD could be accurately assessed without segmenting the whole EAT. Informed consent was obtained from all patients.

This study was conducted retrospectively, with data extracted from previously acquired imaging. Strict inclusion criteria were applied to ensure consistency and reduce potential confounders.

**Figure 1:**
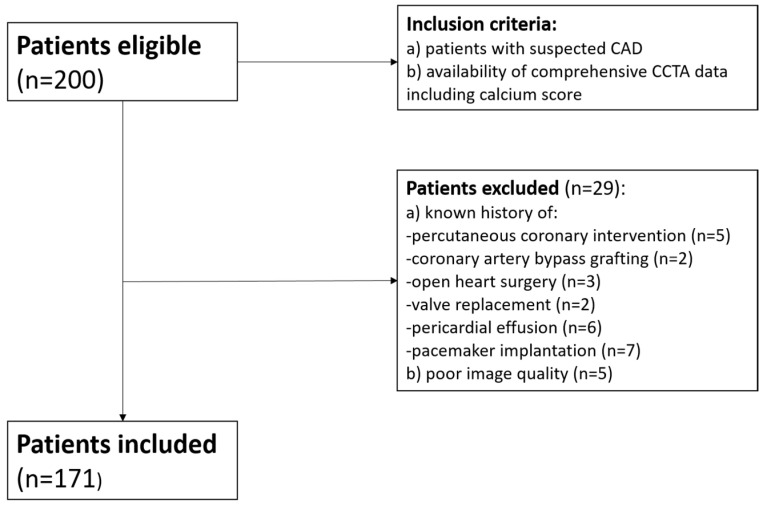
Inclusion and exclusion criteria applied in the study.

Inclusion criteria for patient selection were as follows: (a) suspected CAD; (b) availability of comprehensive CCTA data including calcium score. Exclusion criteria were as follows: known history of percutaneous coronary intervention (PCI), coronary artery bypass grafting (CABG), open heart surgery, valve replacement, pericardial effusion, and those with pacing leads; poor image quality (Figure 1).

### 2.2. Scan Protocol

CCTA examinations were performed using a 256-row multidetector CT scanner (GE Revolution, GE Healthcare, Milwaukee, WI, USA). Nitrates were sublingually administered to all patients, after exclusion of contraindications, to induce vasodilation (Trinitrine, Natispray 0.3 mg). Patients with a heart rate > 75 bpm were treated with intravenous beta-blocker (metoprolol tartrate, Seloken 5 mg). S calcium channel blocker was used if there was contraindication to use b blockers. Prospective gated ECG-triggered non-contrast scan was acquired for coronary calcium scoring with following parameters: tube voltage of 120 kVp, tube current of 185 mAs, detector collimation of 0.625 mm, gantry rotation time of 0.28 s, pitch factor of 0, matrix size of 512 × 512 pixel, section thickness of 2.5 mm, and a Z-plane coverage of 16 cm enabling the imaging of the heart in 1 heartbeat. CCTA images were acquired using a prospective ECG-gated protocol with the following scan parameters: tube voltage of 100 kVp, detector collimation of 0.625 mm, gantry rotation time of 0.28 s, matrix size of 512 × 512 pixel, section thickness of 0.625 mm, and a Z-plane coverage of 16 cm the imaging of the heart in 1 heartbeat. All patients received intravenously 50 mL of iodine contrast medium (400 mg I/mL Iomeprol, Iomeron 400; Bracco Imaging, Milan, Italy) at flow rate of 5 mL/s through an 18-gauge antecubital access by using an automated injector, followed by saline chaser bolus of 30 mL at flow rate of 5 mL/s. Scan delay was determined using a bolus-tracking technique; images were acquired 6 s after the trigger attenuation threshold (150 HU) was reached into a ROI placed in the ascending aorta at the level of pulmonary arteries.

### 2.3. Images Analysis

Three radiologists with different experiences in CCTA analyzed the images to assess global EFD. Discrepancies in segmentation were resolved through consensus.

**Figure 2:**
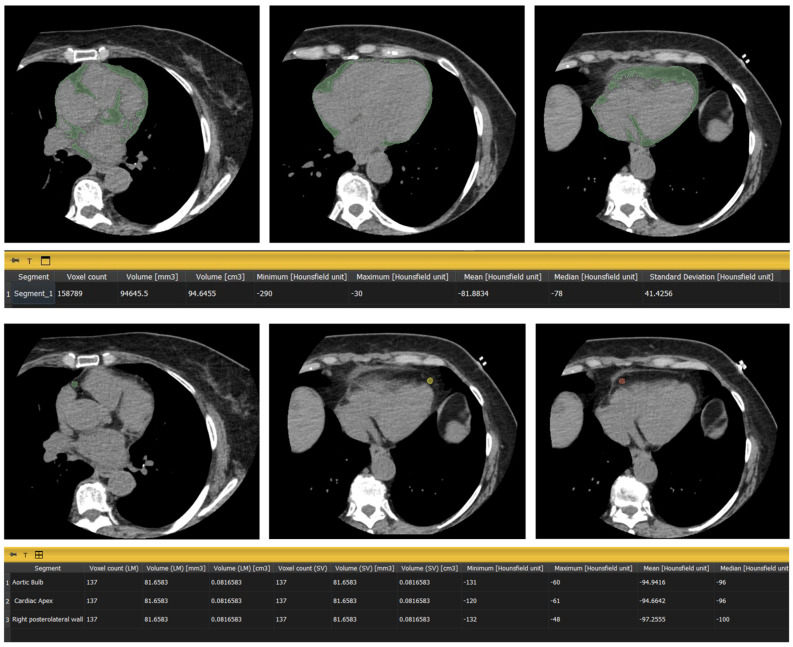
Example of epicardial fat analysis using 3D Slicer; complete segmentation of the total EFV and delineation of three distinct ROIs.

Global EFD was quantified on non-contrast scans acquired for coronary calcium scoring using dedicated free and open source-software (3D Slicer 5.6.2). The software semi-automatically segmented the epicardial fat tissue based on predefined density thresholds (−190 to −30 HU) [17]. For regional EFD analysis, three specific round-shaped ROIs were manually delineated on axial CT slices, each with a surface area of 20 mm^2^, when feasible. The ROIs were positioned at the following levels: (a) aortic bulb, defined as the region surrounding the ascending aorta at the level of the sinuses of Valsalva; (b) right posterolateral wall, located on the lateral aspect of the right ventricle near the right atrioventricular groove; (c) cardiac apex, defined as the most distal portion of the left ventricle. Each ROI was manually delineated to ensure they were positioned away from coronary vessels or other structures such as fibrous bands, and to avoid confounding the fat density measurements. Disagreements in ROI segmentation were addressed through mutual agreement. The mean HU value within each ROI was calculated using 3D Slicer (Figure 2).

### 2.4. Statistical Analysis

Statistical analysis was performed using SPSS software (version 26.0, SPSS Inc., Armonk, NY, USA). Descriptive statistics, including media and standard deviations, were used to summarize the data. The Shapiro–Wilk test was conducted to evaluate the data distribution. Paired t-tests were applied to compare the differences between the EFD values across the entire epicardium and the three ROIs. *p* values less than 0.05 were considered statistically significant. Statistical significance is closely related to clinical importance: statistically significant differences (<0.05) in the ROI may show areas affected by cardiovascular diseases and, therefore, areas that need more attention in clinical practice. The ROI values could be useful in the future to predict the risk of heart diseases and/or to choose personalized treatment.

## 3. Results

A total of 171 patients met the inclusion criteria out of the 200 patients retrospectively enrolled. The study population had a mean age of 62.34 ± 14.73 years, ranging from 17 to 88 years. The number of males and females in the study was 108/171 (63.16%) and 63/171 (36.84%), respectively.

The mean EFD measured for the entire epicardium was −83.92 ± 5.19 HU. This global fat density reflects the average attenuation of fat across the entirety of the epicardium and serves as a baseline against which regional EFD values can be compared.

**Figure 3:**
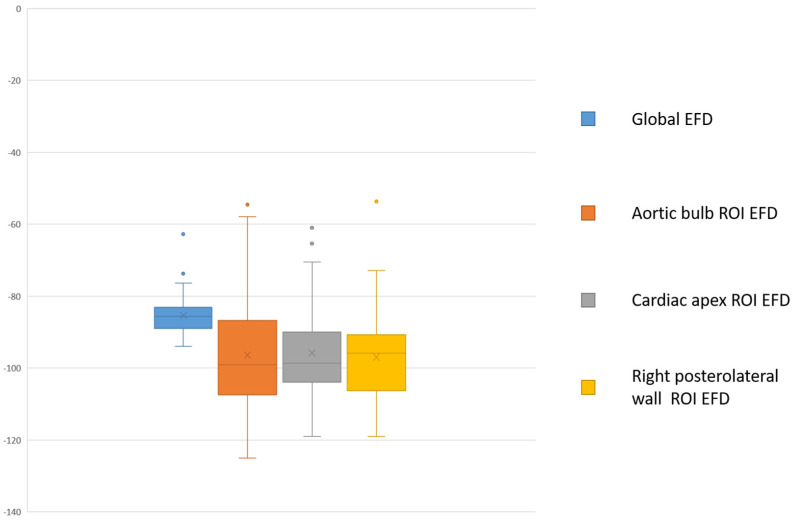
Box plot of mean EFD values calculated globally, at the aortic bulb region, at the apex and at the right posterolateral wall.

The mean EFD values calculated at the aortic bulb region, at the apex, and at the right posterolateral wall were, respectively, −97.54 ± 12.80 HU, −93.42 ± 18.94 HU, and −94.99 ± 12.16 (Figure 3).

The placement of the ROI was not feasible at the level of the bulb and the posterolateral wall in 1 and 5 cases, respectively, due to the limited EFV. 

**Figure 4:**
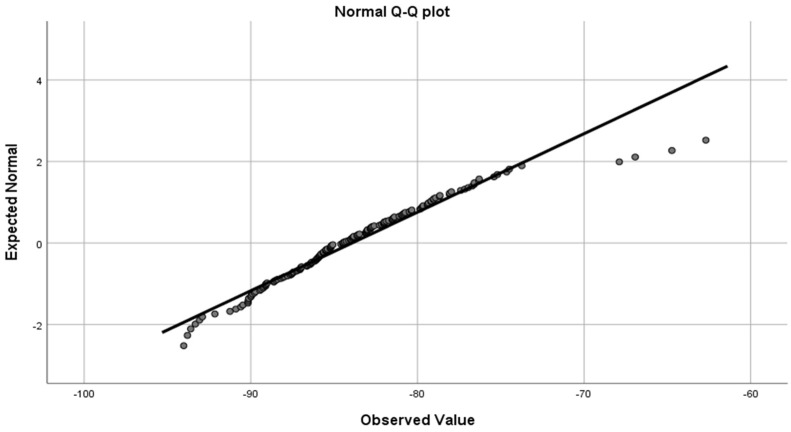
Normal Q-Q plot shows that data can be considered normally distributed.

The Shapiro–Wilk test result was 0.948 (*p*-value = 0.1) (Figure 4), meaning the data can be considered normally distributed. 

Analysis of the three predefined ROIs revealed significant regional variability in EFD values. 

The mean difference between global EFD and bulb ROI EFD is 13.63 (±11.42), with a mean standard error of 0.88. The confidence interval (95%) ranges from 11.90 to 15.36, indicating that the difference is statistically significant. The *t*-value of 15.564 and the *p*-value of <0.000 confirm a highly significant difference between these two measurements. 

The comparison between global EFD and apex ROI EFD revealed a slightly smaller mean difference of 9.50 ± 17.72, with a mean standard error of 1.36. The 95% confidence interval spans from 6.83 to 12.18, demonstrating significant variability but still a statistically significant difference with the *t*-value of 7.011 and *p*-value of <0.000. 

For the comparison between global EFD and posterolateral wall ROI EFD, the mean difference is 11.04 (±10.41), with a mean standard error of 0.81. The confidence interval (95%) ranges from 9.45 to 12.64. This difference is also statistically significant, supported by a *t*-value of 13.672 and a *p*-value of <0.000. 

Paired *t*-tests results are resumed in Table 1.

## 4. Discussion

The interest in EAT is increasing in the scientific community as a possible non-invasive biomarker for cardiovascular risk [18]. EAT has dishomogeneous distribution around the heart: most are located at the base and apex of the heart, in the cardiac sulci, and around the coronary arteries; it is also thicker around the right ventricle than the left ventricle [19]. Several studies have demonstrated that pericoronary fat density, measured in Hounsfield Units (HU), is associated with the presence and severity of coronary artery disease (CAD). Higher pericoronary fat attenuation has been linked to increased vascular inflammation, which in turn correlates with greater plaque burden and the presence of high-risk plaques. Specifically, regions adjacent to significant stenotic lesions tend to show elevated fat density, likely due to the local inflammatory response associated with atherosclerotic progression. In contrast, non-stenotic or mildly diseased segments often exhibit lower fat attenuation, suggesting less inflammatory activity. Furthermore, fat density changes dynamically in response to plaque morphology; for instance, vulnerable plaques with a lipid-rich necrotic core and positive remodeling tend to be surrounded by higher-density pericoronary fat compared to more stable calcified plaques.

Numerous studies in the literature revealed that the increase in EFV and EAT’s alterations are related to CAD, atherosclerosis, and cardiac arrhythmias [20]. When the EF volume increases, it becomes hypoxic and dysfunctional, increases lipolysis and consequent inflammation which eventually results in a shift in its metabolic profile and alters the homeostasis.

A more precise correlation is represented by EFD: it describes the composition of EAT and its alteration is often related to inflammation, fibrosis, and infiltration of the connective tissues [21,22]. The use of non-invasive imaging methods for evaluating EFD, such as CCT, represents an important innovation for monitoring cardiovascular risk.

EAT functions as fatty tissue and it has an endocrine effect. Alterations of EAT promote the secretion of adipokine. In pathological conditions, it can promote the secretion of pro-inflammatory adipokine, reactive oxygen species, oxidative stress, and even autophagy, thus affecting cardiac function [23].

The quantification of EAT has traditionally relied on imaging modalities such as echocardiography, CCT, and CMR. Among these, CCT is considered the gold standard for assessing both the volume and density of EAT, offering high spatial resolution and the ability to quantify fat density using HU. However, quantitative evaluation of EFD is still limited by the need for complex segmentation software and the difficulty in obtaining repeatable and accurate measurements in daily clinical practice [14].

In this regard, our study focused attention on the possibility of evaluating EFD in an alternative and more accessible way compared to complete segmentation. The alternative method proposed in this study was a regional evaluation through predefined ROI, of pre-established dimensions (20 mm^2^) already used in the literature [24]. ROIs are easier to use and permit repeatable and accurate measurements.

Regional analysis of EFD through ROI would be very simple and useful. Through the ROIs, it would be easy to follow any alterations of EFD and therefore evaluate changes in the cardiovascular risk of each patient. The use of ROIs could be useful for the follow-up of patients with cardiovascular risk and to modify their therapy based on their cardiovascular risk condition.

CT scans for calcium score were used to position ROI at specific points:

1. Aortic bulb: The region at the base of the heart near the aortic root, chosen for its proximity to the aortic valve and proximal segments of coronary arteries, as well as its exposure to high mechanical stress; this region is critical due to its proximity to the coronary ostia and its role in hemodynamic regulation. The fat in this area is highly influenced by shear stress and systemic inflammation, factors known to contribute to the development and progression of CAD.
2. Right posterolateral wall: A region located on the right side of the heart, chosen for its relative distance from major coronary vessels and its association with arrhythmogenesis. The fat in this area is less exposed to high shear stress and its proximity to major venous structures, including the coronary sinus, could influence the local adipose tissue environment, making it an interesting site for evaluating differences in fat attenuation compared to more hemodynamically active regions.
3. Cardiac apex: The most distal part of the heart, located inferiorly and anteriorly. The fat in this location is less exposed to direct vascular inflammation from major coronary arteries, allowing for a comparison of regional variations in fat density. This approach may help to differentiate systemic inflammatory effects from localized vascular inflammation [14,25].

In our study, we used scans acquired for coronary calcium scoring. These scans were primarily used for screening purposes, as they identify calcifications of the coronary arteries, which are indicative of a patient’s risk for cardiovascular events. 

The absence of contrast agents ensures a more stable and accurate measurement of tissue density in HU, as contrast can alter tissue intensity and compromise the consistency of these measurements. 

Calcium scoring scans are also performed with lower radiation doses compared to contrast-enhanced cardiac CT, making them safer for repeated use. Furthermore, they are generally more cost-effective due to the simpler procedure and absence of contrast material.

Our results showed that EFD varies significantly between different regions of the heart and across the three ROIs. The mean EFD for the entire epicardial fat is −84.75 ± 4.31 HU, in line with the values reported in the literature, while regional measurements in the three predefined ROIs, i.e., aortic bulb, right posterolateral wall, and cardiac apex, showed significant regional variability in the posterolateral wall and cardiac apex, but not at the level of the aortic bulb.

These regional differences demonstrate the distribution of EAT. More EAT is in the base of the heart (including the aortic bulb) and apex, but the right posterolateral wall, which is associated with greater mechanical activity and stress [20], also has a higher density than other areas. The variability in EFD values between regions suggests that some areas of the heart are more susceptible to infiltration of connective tissues or inflammatory processes and, therefore, more susceptible to cardiovascular pathologies. Regional differences in EFD may indicate localized inflammatory activity, which could influence the progression of atherosclerosis as plaque vulnerability, coronary artery stenosis, and visual plaque burden. The paired *t*-test results further emphasize this variability, underscoring their potential clinical relevance in assessing disease progression and cardiovascular risk, paving the way for future research. In addition, heterogeneity in EFD distribution, particularly around the atria, may contribute to atrial remodeling and fibrosis, potentially playing a role in the development of arrhythmias such as atrial fibrillation [10,11,12,13,21,22].

The variation in EFD between different ROIs could also be linked to differences in cardiac function and metabolic responses. The EFD measured at the aortic bulb was lower than the values measured at the other two regions (the apex and the right posterolateral wall). This could indicate that the fat surrounding the aortic bulb is more lipid-rich and less fibrotic or inflamed compared to the other regions. The aortic bulb is a region of high hemodynamic stress, which explains why the fat in this area is more metabolically active and less subjected to pathological changes such as fibrosis or inflammation.

Although measuring EFD using CCT is still difficult due to the necessity to use complex software and long segmentation times, the ROI-based approach, as demonstrated in this study, could offer a practical and more easily applicable solution in clinical settings.

Our study has some limitations. First of all, the relatively small size of our sample could limit generalization of the results. Studies on a larger sample and histological validation are needed to confirm these results and to establish more precise cutoff values for regional EFD measurements. Another limitation of this study is its retrospective design, which may introduce selection bias and limit the generalizability of the findings. Additionally, while the study demonstrated significant regional variability in EFD, it did not explain how these variations correlate with clinical outcomes. Future research should investigate if specific regional EFD values are predictive of cardiovascular events, such as myocardial infarction, heart failure, or atrial fibrillation. A potential limitation of this study is the lack of a formal assessment of inter-observer variability in ROI placement. Given the manual nature of this method, some degree of variability is expected, which could impact reproducibility. Future studies should include systematic evaluations of observer agreement to further validate this approach.

In the future, studies with larger sample sizes and advanced imaging techniques—such as the use of artificial intelligence in more accurate segmentation and measurement of fat density—could enhance the use of EFD as a predictive biomarker for cardiovascular disease.

Additionally, monitoring changes in EFD over time could provide valuable insights into the mechanisms of cardiovascular disease progression and the effectiveness of specific therapies. To achieve a more accurate and comprehensive understanding of regional EFD’s predictive value for conditions like heart failure and myocardial infarction, and to establish it as an early biomarker, larger-scale studies are essential. 

## 5. Conclusions

This study highlights significant regional variability in EFD across specific cardiac regions, providing insights into the feasibility and limitations of ROI-based EFD assessment. The observed differences between regions, such as lower EFD at the aortic bulb and higher values at the apex and right posterolateral wall, indicate that localized measurements may not reliably reflect global epicardial fat properties.

To overcome these limitations, accurate EFD evaluation requires dedicated segmentation software capable of analyzing the entire epicardium. Such tools are essential for achieving precise and consistent measurements, which are crucial for integrating EFD into cardiovascular risk assessment and routine clinical practice.

## Figures and Tables

**Table 1:** Paired *t*-tests results for the comparison between global EFD and regional ROIs.

	Mean	S.D.	Mean Standard Error	Confidence Interval 95%		*t*	gl	*p* Value
				Inferior	Superior			
global EFD—bulbROI EFD	13.63155	11.41929	0.87582	11.90260	15.36051	15.564	169	0.000
global EFD—apex ROI EFD	9.50123	17.72049	1.35512	6.82620	12.17626	7.011	170	0.000
global EFD—postero-lateral wall ROI EFD	11.04425	10.40752	0.80778	9.44933	12.63917	13.672	165	0.000

## Data Availability

The raw data supporting the conclusions of this article will be made available by the authors on request.

## Abbreviations

The following abbreviations are used in this manuscript:

CCT	Cardiac computed tomography
EAT	Epicardial adipose tissue
EFT	Epicardial fat tissue
EFD	Epicardial fat density
PAT	Pericardial adipose tissue
CAD	Coronary artery disease
HU	Hounsfield units
CMR	Cardiac magnetic resonance
CCTA	Coronary CT angiography
ROI	Region of interest
PCI	Percutaneous coronary intervention
CABG	Coronary artery bypass grafting
